# Supplementary material for: Cost-effectiveness modelling to optimise active screening strategy for gambiense human African trypanosomiasis in endemic areas of the Democratic Republic of Congo
Source: BMC Med. 2021 Apr 1;19:86. doi: 10.1186/s12916-021-01943-4 (PMC8017623; doi:10.1186/s12916-021-01943-4)
Supplement: Supplementary file 1 — Additional file 1 Figures S1–S10 and Tables S1–S7. Supporting information for the manuscript including further details of the gHAT modelling, explanations and derivations of all net monetary benefit parameters, a short analysis of a different health zone, details on reactive screening, some additional results, and a table of how we satisfied the five principles of the Neglected Tropical Diseases Modelling Consortium. Figure S1 – Schematic of the compartmental model for gHAT infection dynamics in humans (low- and high-risk) and tsetse. Figure S2 – Histograms of screening coverage for (A) Kwamouth and (B) Mosango. Figure S3 – The cost-effectiveness of active screening strategies for the health zone of Mosango. Figure S4 – The effect of the number of zero-detections in reactive screening on the cost effective acceptability curves (CEACs). Figure S5 – Active and reactive screenings information. Figure S6 – The time interval between observing a passive detection and resuming reactive screening for different values of active zero-detections. Figure S7 – Distributions of (A) the change in cost of an active screening strategy and (B) the number of DALYs averted for 30- and 100-year time-horizons. Figure S8 – Theoretical optimum strategy for mean simulation of infection dynamics given WTP and pt. Figure S9 – Mean change in costs relative to the assumed interventions for a village with 55% screening coverage annually with 3 zero-detections. Figure S10 – Linear fits through the results of different active screening strategies from Fig. 5. Table S1 – The transition rates of the Markov-chain gHAT-infection model and additional ordinary differential equation component of the model. Table S2 – Parameter notation and values for the gHAT infection model. Table S3 – Net monetary benefit parameters that determine our manuscript parameters. Table S4 – Proportion of people receiving each type of treatment. Table S5 – Formulation of manuscript parameters from cost data. Table S6 – The va [file 12916_2021_1943_MOESM1_ESM.pdf]

Appendix: Cost-effectiveness modelling to optimise active screening strategy for *gambiense* human African trypanosomiasis in the Democratic Republic of Congo

Christopher N. Davis[1,2], Kat S. Rock[1,2], Marina Antillón[3,4]
Erick Mwamba Miaka[5], Matt J. Keeling[1,2,6]

* Corresponding author: C.Davis.7@warwick.ac.uk
1 Mathematics Institute, University of Warwick, Coventry, CV4 7AL, UK
2 Zeeman Institute (SBIDER), University of Warwick, Coventry, CV4 7AL, UK
3 Swiss Tropical and Public Health Institute, Socinstrasse 57, 4051 Basel, Switzerland
4 University of Basel, Petersplatz 1, 4051 Basel, Switzerland
5 Programme National de Lutte contre la Trypanosomiase Humaine Africaine (PNLTHA), Ave Coisement Liberation et Bd Triomphal No 1, Commune de Kasavubu, Kinshasa, DRC
6 School of Life Sciences, University of Warwick, Coventry, CV4 7AL, UK

The gHAT model

We use a mechanistic compartmental model for gHAT infection that outputs the number of humans and the proportion of the total number of vectors in each infection compartment. Humans are defined as: susceptible S_H, exposed (but not infectious) E_H, Stage 1 infection I_{1H}, Stage 2 infection I_{2H} and hospitalised (or recovering at home) R_H. The vectors (tsetse) are: in the pupal stage P_V, teneral (unfed) S_V, infected and in their extrinsic incubation period (there are three classes to create a gamma distributed period) E_V, infectious tsetse I_V, and non-teneral (fed) but uninfected tsetse G_V. The population size is assumed to be constant and thus $N_{Hj} = S_{Hj}(t) + E_{Hj}(t) + I_{1Hj}(t) + I_{2Hj}(t) + R_{Hj}(t)$, $j = 1, 2$, where $j = 1$ for low-risk individuals, randomly participating in active screening, and $j = 2$ for high-risk individuals, never participating in active screening, each at time $t > 0$.

Humans can become exposed, E_H, upon a bite from an infective tsetse and progress to Stage 1 infection, I_{H1}, before either moving to Stage 2 infection I_{H2} followed by the non-infectious class R_H, or moving directly to the non-infectious class, if the infection is detected early. Stage 1 detection is at rate $\eta_H(Y) = \eta_H^{\text{post}}(\eta_{H\text{amp}}/(1+\exp(-d_{\text{steep}}(t-d_{\text{change}}))))$ and Stage 2 is at rate $\gamma_H(Y) = \gamma_H^{\text{post}}(\gamma_{H\text{amp}}/(1+\exp(-d_{\text{steep}}(t-d_{\text{change}}))))$. The birth rates B_{Hi} are given by $\mu_H N_{Hi}$ for $i = 1, 2$ and B_V is equal to $\mu_V N_H$, where the different numbers of humans and vectors are accounted for in the effective tsetse density m_{eff}. Active screening moves exposed or infected people directly to the hospitalised class and is simulated by randomly selecting from the low-risk population with 91% probability of true positives being detected (the sensitivity of the screening algorithm). Specificity of the algorithm is taken as 99.91%.

If tsetse progress from the pupal stage P_V, the unfed fly can become exposed and infected, E_V and I_V, on consumption of a blood-meal, a proportion of which are taken on humans. Alternatively, first blood-meals not resulting in exposure means tsetse are less susceptible to trypanosomes in future meals, G_V. We define $\lambda_V = \alpha p_V(f_{H1}\frac{I_{1H1}+I_{2H1}}{N_{H1}} + f_{H2}\frac{I_{1H2}+I_{2H2}}{N_{H2}})$ as the rate of exposure, where $f_{H1} = k_1/(k_1 + rk_2)$ and $f_{H2} = rk_2/(k_1 + rk_2)$, such that $f_{H1} + f_{H2} = f_H$. Infected animals are not considered.

The compartments of the model and possible transitions between them are shown graphically in Figure S1. The transmission of infection between humans and tsetse is shown by grey paths. Transition rates of the Markov-chain model are defined explicitly in Table S1.

Table S2 defines the model parameters used in the model for HAT infection dynamics. These parameter values are taken from Crump *et al.* [30], which were either sourced from literature, where well-defined, or otherwise (in the case of R_0, k_1, k_2, r, u, Se, η_H^{post}, $\eta_{H\text{amp}}$, γ_H^{pre}, γ_H^{post}, $\eta_{H\text{amp}}$, d_{change}, and d_{steep}) taken as the median of the distribution obtained by model fitting using a Metropolis–Hastings MCMC algorithm that matched the deterministic version of the model to incidence data from the WHO HAT Atlas [40, 41].

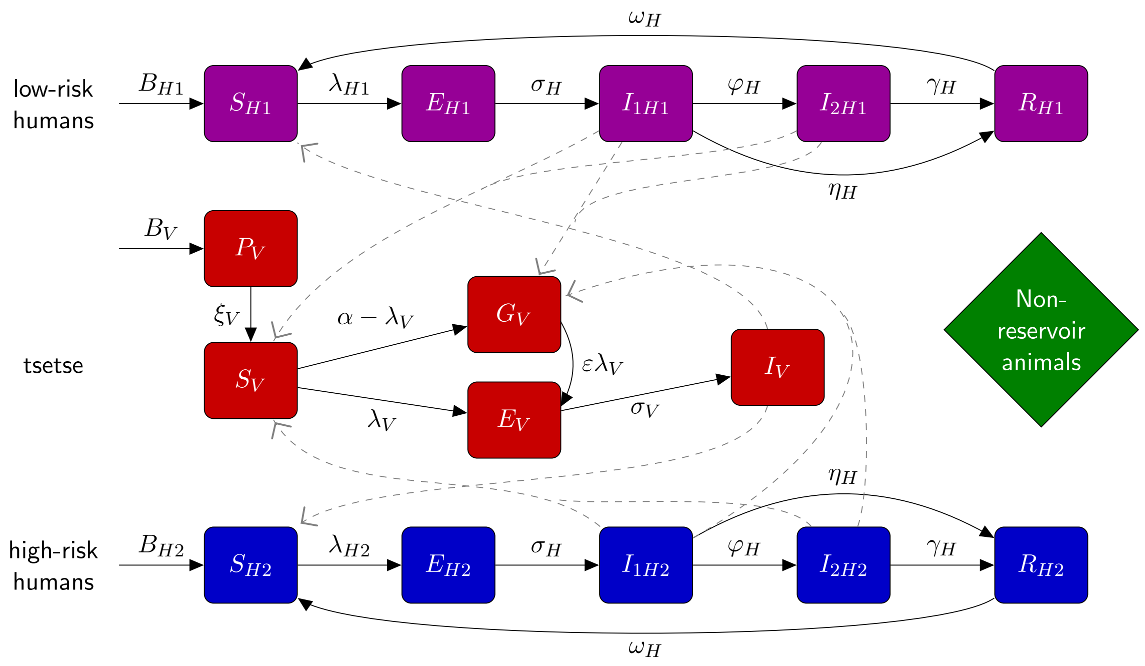

Figure S1: Schematic of the compartmental model for gHAT infection dynamics in humans (low- and high-risk) and tsetse. Adapted from Crump *et al.* [30].

Table S1: The transition rates of the Markov-chain gHAT-infection model and additional ordinary differential equation component of the model.

Event	Transition	Rate
Recovery from hospitalisation	$S_{Hj} \to S_{Hj}+1, R_{Hj} \to R_{Hj}-1$	$\omega_H R_{Hj}$
Natural death of hospitalised	$S_{Hj} \to S_{Hj}+1, R_{Hj} \to R_{Hj}-1$	$\mu_H R_{Hj}$
Exposure of susceptibles	$S_{Hj} \to S_{Hj}-1, E_{Hj} \to E_{Hj}+1$	$f_{Hj}\alpha m_{\text{eff}} S_{Hj} I_V / N_{Hj}$
Progression to Stage 1 infection	$E_{Hj} \to E_{Hj}-1, I_{1Hj} \to I_{1Hj}+1$	$\sigma_H E_{Hj}$
Natural death of exposed	$S_{Hj} \to S_{Hj}+1, E_{Hj} \to E_{Hj}-1$	$\mu_H E_{Hj}$
Progression to Stage 2 infection	$I_{1Hj} \to I_{1Hj}-1, I_{2Hj} \to I_{2Hj}+1$	$\phi_H I_{1Hj}$
Natural death of Stage 1 infection	$S_{Hj} \to S_{Hj}+1, I_{1Hj} \to I_{1Hj}-1$	$\mu_H I_{1Hj}$
Passive treatment from Stage 1 infection	$I_{1Hj} \to I_{1Hj}-1, R_{Hj} \to R_{Hj}+1$	$\eta_H(Y) I_{1Hj}$
Passive treatment or death from Stage 2 infection	$I_{2Hj} \to I_{2Hj}-1, R_{Hj} \to R_{Hj}+1$	$\gamma_H(Y) I_{2Hj}$
Natural death of Stage 2 infection	$S_{Hj} \to S_{Hj}+1, I_{2Hj} \to I_{2Hj}-1$	$\mu_H I_{2Hj}$
Infection importation	$S_{Hj} \to S_{Hj}-1, E_{Hj} \to E_{Hj}+1$	δS_{Hj}

$$
\begin{aligned}
\frac{\mathrm{d}P_V}{\mathrm{d}t} &= B_V N_V - (\xi_V + \frac{P_V}{K})P_V \\
\frac{\mathrm{d}S_V}{\mathrm{d}t} &= \xi_V \mathbb{P}(\text{pupating}) P_V - \alpha S_V - \mu_V S_V \\
\frac{\mathrm{d}E_{1V}}{\mathrm{d}t} &= \alpha p_V (f_{H1}\frac{I_{1H1}+I_{2H1}}{N_{H1}} + f_{H2}\frac{I_{1H2}+I_{2H2}}{N_{H2}})(S_V + \varepsilon G_V) - (3\sigma_V + \mu_V)E_{1V} \\
\frac{\mathrm{d}E_{2V}}{\mathrm{d}t} &= 3\sigma_V E_{1V} - (3\sigma_V + \mu_V)E_{2V} \\
\frac{\mathrm{d}E_{3V}}{\mathrm{d}t} &= 3\sigma_V E_{2V} - (3\sigma_V + \mu_V)E_{3V} \\
\frac{\mathrm{d}I_V}{\mathrm{d}t} &= 3\sigma_V E_{3V} - \mu_V I_V \\
\frac{\mathrm{d}G_V}{\mathrm{d}t} &= \alpha(1 - p_V(f_{H1}\frac{I_{1H1}+I_{2H1}}{N_{H1}} + f_{H2}\frac{I_{1H2}+I_{2H2}}{N_{H2}}))S_V \\
&\quad - \alpha p_V (f_{H1}\frac{I_{1H1}+I_{2H1}}{N_{H1}} + f_{H2}\frac{I_{1H2}+I_{2H2}}{N_{H2}})\varepsilon G_V - \mu_V G_V
\end{aligned}
$$

Table S2: Parameter notation and values for the gHAT infection model.

Parameter	Description	Value	Source
μ_H	Natural human mortality rate	5.4795×10^{-5} days^{-1}	[31]
ω_H	Human recovery rate	0.006 days^{-1}	[32]
σ_H	Human incubation rate	0.0833 days^{-1}	[33]
ϕ_H	Stage 1 to 2 progression	0.0019 days^{-1}	[34, 35]
η_H^{post}	Post-1998 exit rate from Stage 1 by treatment or death	0.00012 days^{-1}	[30]
$\eta_{H\text{amp}}$	Maximum increase in the Stage 1 exit rate	2.51	[30]
γ_H^{pre}	Pre-1998 exit rate from Stage 2 by treatment or death	0.0017 days^{-1}	[30]
γ_H^{post}	Post-1998 exit rate from Stage 2 by treatment or death	0.0019 days^{-1}	[30]
$\gamma_{H\text{amp}}$	Maximum increase in the Stage 2 exit rate	0.514	[30]
d_{steep}	Steepness of the improvement rates for Stage 1 and Stage 2 passive detection	0.941	[30]
d_{change}	Switching year of the improvement rate for Stage 1 and Stage 2 passive detection	2006	[30]
N_H	Human population size	Varies	N/A
μ_V	Tsetse mortality rate	0.03 days^{-1}	[33]
σ_V	Tsetse incubation rate	0.034 days^{-1}	[36, 37]
ε	Reduced non-teneral susceptibility factor	0.05	[30]
α	Tsetse bite rate	0.333 days^{-1}	[5]
m_{eff}	Effective tsetse density	6.56	[30]
p_V	Probability of tsetse infection per single infective bite	0.065	[33]
f_H	Proportion of blood-meals on humans	0.09	[38]
K	Pupal density dependence	111.09	[24]
$\mathbb{P}$(pupating)	Probability of pupating	0.75	[24]
ξ_V	Pupal death rate	0.037	[24]
B_V	Total deposit rate	0.0505	[24]
Se	Sensitivity	0.91	[39]
Sp	Specificity	0.9991	[30]
k_1	Proportion of low-risk humans	0.902	[30]
k_2	Proportion of high-risk humans	0.098	[30]
r	Relative bites taken on high-risk humans compared to low-risk	6.61	[30]
u	Reporting probability for Stage 2 cases	0.2737	[30]
δ	Importation of infection rate	3.4×10^{-6} days^{-1}	[26]

Net monetary benefit parameters

In developing a parameterised cost function for active screening strategies, we have ensured transferability of costs across time by updating all values to 2018 US dollars (USD). All costs are converted to local currency units in the year of the study, inflated with the consumer price index (CPI) to find the 2018 values, and then converted to 2018 USD with the exchange rate. We also ensure transferability of costs across settings, by following the 2003 WHO Guide to Cost-effectiveness recommendations [42]. For non-traded items (nurse and doctor time) we turn the USD or local currency unit (LCU) prices into PPP (international dollars) in the year of the cost study and then turn the value in international dollars to local currency (still in the year of the study) of the country where a cost estimate is needed. Then, we use the CPI to inflate costs to 2018 levels and then use the exchange rate to convert to 2018 USD values.

Table S3 provides a list of component parameters determined from the literature, along with a source. Table S4 shows the proportion of people given each treatment. Table S5 converts these component parameters and treatment proportions into the net monetary benefit parameters seen in the main manuscript. All NMB parameters are taken as fixed values in this study.

All parameters were obtained by an extensive literature search, which were then converted, as described above, into 2018 USD. Some justifications for these calculations are given below.

Table S3: Net monetary benefit parameters that determine our manuscript parameters. All values are assumed fixed. All costs are in 2018 USD.

Parameter	Description	Value	Source
D_{death}	Age of death from infection	27 years	[43]
D_{life}	Life expectancy	60.21 years	[44]
D_{stage1}	Disability weight – Stage 1	0.133	[45]
D_{stage2}	Disability weight – Stage 2	0.5432	[45]
N_{cap}	Active screening capacity per year	60,000	[11]
C_{cap}	Capital costs of a traditional active control team	$11,406	[46]
C_{rec}	Recurrent costs of a traditional active control team	$40,000	[46]
C_{catt}	CATT algorithm cost	$0.54	[11, 47]
M_{wasa}	CATT wastage in active screening mark up	1.08	[46]
M_{wastep}	CATT wastage in passive surveillance mark up	1.25	[48]
C_{rdt}	RDT algorithm cost	$1.41	[11]
C_{mic1}	Microscopy (blood sample, LNA, mAECT)cost	$9.53	[48]
C_{mic2}	Microscopy (blood sample, LNA, CTC, mAECT) cosr	$11.30	[48]
C_{lumb}	Lumbar puncture and laboratory exam cost	$23.02	[49]
C_{out}	Outpatient consultation cost	$0.43	[50, 51]
C_{in}	Hospital day cost	$1.40	[50, 51]
C_{pent}	Course of pentamidine cost	$54.00	[52]
L_{pent}	Length of hospital stay for pentamidine	7 days	[53]
C_{nect}	Course of NECT cost	$460.00	[54]
L_{nect}	Length of hospital stay for NECT	10 days	[54]
C_{fex}	Course of fexinidazole cost	$50.00	[55]
L_{fex}	Length of hospital stay for fexinidazole	10 days	[54]
M_{del}	Drug delivery mark up	1.45	[50, 51]
M_{prog}	National programme mark up	1.15	[46]

Table S4: Proportion of people receiving each type of treatment. Values are taken from Antillon *et al.* [56].

Parameter	Treatment	Value
Stage 1		
P_{pent1}	Pentamidine	0.06
P_{fexin1}	Fexinidazole-inpatient	0.02
$P_{fexout1}$	Fexinidazole-outpatient	0.92
Stage 2		
P_{nect2}	NECT	0.65
P_{fexin2}	Fexinidazole-inpatient	0.01
$P_{fexout2}$	Fexinidazole-outpatient	0.34

Table S5: Formulation of manuscript parameters from cost data.

Parameter	Name	Formulation
D_1	Discounted average years of life lost per death	$D_{life} - D_{death}$
D_2	Stage 1 disability weight	D_{stage1}
D_3	Stage 2 disability weight	D_{stage2}
C_1	Active screening capital cost per person	$C_{cap}M_{prog}/N_{cap}$
C_2	Active screening recurrent cost per person	$C_{rec}M_{prog}/N_{cap}$
C_3	Active screening test per person	$C_{catt}M_{del}M_{wastea}M_{prog}$
C_4	Confirmation per person	$C_{mic1}M_{prog}$
C_5	Stage determination per person in Stage 1	$P_{pent1}C_{lumb}M_{prog}$
C_6	Stage determination per person in Stage 2	$P_{nect2}C_{lumb}M_{prog}$
C_7	Passive screening person in Stage 1	$(C_{catt}M_{del}M_{wasp} + C_{out} + C_{mic1} + P_{pent1}C_{lumb})M_{prog}$
C_8	Passive screening person in Stage 2	$(C_{catt}M_{del}M_{wasp} + C_{out} + C_{mic1} + P_{nect2}C_{lumb})M_{prog}$
C_9	Stage 1 treatment per person	$(P_{pent1}(C_{pent}M_{del} + C_{in}L_{pent}) + P_{fexin1}(C_{fex}M_{del} + C_{in}L_{fex}) + P_{fexout1}(C_{fex}M_{del} + C_{out}))M_{prog}$
C_{10}	Stage 2 treatment per person	$(P_{nect2}(C_{nect}M_{del} + C_{in}L_{nect}) + P_{fexin2}(C_{fex}M_{del} + C_{in}L_{fex}) + P_{fexout2}(C_{fex}M_{del} + C_{out}))M_{prog}$

Disability weight – Stage 1 & 2, D_{stage1} & D_{stage2}

The Global Burden of Disease listed the impact of sleeping sickness as equivalent to the health state labeled 'Motor plus cognitive impairments, severe' with a given estimated disability weight of 0.542, using the 2013 weight values. No distinction was made between Stage 1 and 2 of the disease. While this seems appropriate for the second stage of sleeping sickness, for Stage 1 disability we chose to use the disability weights for equivalent to 'Infectious disease, acute episode, severe', which is described as 'has a high fever and pain, and feels very weak, which causes great difficulty with daily activities' and has a much lower disability weight equivalent to 0.133.

It should be noted that other cost-effectiveness analyses have used different values for disability weights [11,55,57]. These values arise from the 1994 Global Burden of Disease Study but we prefer to consider updated values. Since most of the disability is due to deaths, rather than illness during life, we do not believe that this difference is cause for concern.

Active screening capacity per year, N_{cap}

The capacity of an active screening team in DRC N_{cap}, has a mean of 60,000 and a lower bound of 40,000 [47] and an upper bound of 70,000 with a work year of 220 days, according to ITM-Antwerp; capacity can vary by location. Teams are managed by the coordination and serve a span of multiple health zones and dozens of villages.

Capital costs of a traditional active control team, C_{cap} & Recurrent costs of a traditional active control team, C_{rec}

Lutumba *et al* [47] calculated the total cost of screening 40,000 patients in 2003 was 46,734 Euros (1 Euro = 0.86 USD in 2003). Of that value, 21% of the costs were capital costs, 8216.99 in 2018 USD, with the remainder recurrent fixed costs, 30,912 in 2018 USD. The publication did not indicate whether that value was annualised or not.

According to [11], the cost for a mobile team that screens 250 patients per day for 220 days a year has capital investments (annualized for five years) of $12,000 for a team that administers CATT and $12,781 for a team that administers RDT (in 2013 USD). Annual recurrent costs of $30,307 and daily recurrent costs of $97 give total recurrent costs of equal 30,717 in 2018 USD.

According to [46], the recurrent cost of an active screening team was $11,406 in 2018 for a team that administer CATT. And the management and recurrent costs of an active screening team was $42,408 in 2018.

In order to be conservative about the cost of a team, we have estimated that the yearly cost is $40,000 for a team that can screen 60,000 people per year. For recurrent costs, we took the estimate by [46] of $11,406.

CATT algorithm cost, C_{catt}

To our knowledge, costs for CATT tests C_{catt}, are only featured in three sources. The cost includes test materials plus the cost of the lancet. [52] list the cost as 0.73 in 1998 USD, equivalent to 0.34 in 2018 USD; [47] give 0.52 in 2003 USD, equivalent to 0.37 in 2018 USD; and [11] listed the cost of CATT test at 0.70 for a mobile team, equivalent to 0.42 in 2018 USD. [46] listed the cost of a CATT test at $0.74 in 2018 USD.

The midpoint of the highest and lowest estimate is $0.54 in 2018 values.

Lumbar puncture and laboratory exam cost, C_{lumb}

Costs for lumbar puncture tests were reported by Snijders and colleagues as 23.02 in 2018 USD. [49] have reported a similar value in Chad, confirming the cost by Snijders and colleagues.

RDT algorithm cost, C_{rdt}

For RDTs, both [55] and [11] coincided on a cost of 0.50 USD 2013 for the test in the international market (after a $0.25 subsidy paid for outside of DRC). [11] also calculated staff costs and shipment for 0.32 USD 2013. Because we can split the cost between tradable and non-tradable costs, we inflate and adjust the costs for the staff costs and shipment and then add the cost of the RDT. In terms of 2018 USD costs, the shipment and the staff costs are 0.19, and the total cost is 0.94. [48] reported a cost between 0.85 and 1.97, depending on the company from which the test is purchased. The midpoint of the range is 1.41, which is the value we have used.

Hospital day cost, C_{in}

In 2010, a consult at a primary hospital in DRC would be $2.41 (0.90, 5.73), a consult at a secondary hospital in DRC would be $2.59 (0.98, 5.81), and a consult at a tertiary hospital in DRC would be $3.25 (1.32, 7.20) in international dollars [50, 51]. To be conservative, we will take the cost of a hospital day in a tertiary hospital, as gHAT is usually a disease where the treatment requires special training. The equivalent mean estimates in 2010 USD is $1.68 per day at a tertiary hospital. After converting to local currency, applying the inflation index, and converting to 2018 USD, $1.40 per day at a tertiary hospital.

Outpatient consultation cost, C_{out}

We got the estimates of outpatient consultation costs from the 2010 WHO CHOICE estimates (recently updated by Bertram 2017 and Stenberg 2018). In 2010, a consultation in DRC at a health post without beds was estimated at 0.81 (0.17, 2.65) I$ and a health post with beds was estimated at 1.00 (0.21, 2.86) I$. To be conservative, we will take the cost of an outpatient consult in a health post without beds, as gHAT is usually a disease where the treatment requires special training.

The equivalent estimates in 2010 USD is 0.53. After converting to local currency, applying the inflation index, and converting to 2018 USD, the estimates are $0.43.

Study health zones — Kwamouth and Mosango

This manuscript considers how to optimise active screening for *gambiense* human African trypanosomiasis for a general endemic health zone within the DRC. However, the specific parameterisation is fit to incidence data from the health zone of Kwamouth. Active screening in Kwamouth has a median screening coverage of 55% for each village-level active screening. The health zone of Mosango in the DRC, which is also in the former Bandundu province, and now lies in the Kwilu province, is another health zone considered traditionally endemic with gHAT. Here, we find a median coverage of 57% for each village active screening. Figure S2 shows histograms of screening coverage from 2000–2016 for these two health zones.

To consider whether the two health zones show different results, we use our gHAT model parameterised to incidence data from this health zone, and produce the same cost-effectiveness results for the realistic strategies. Here we use the median screening coverage of typical screenings from Mosango, which is 57%, along with the model parameters matched health zone.

Qualitatively, we see that the cost-effectiveness plane for Mosango looks similar to Kwamouth, however, since there are fewer reported cases in Mosango, fewer DALYs are averted, and the cost of implementing active screening strategies is lower, as operations are terminated earlier than in Kwamouth (Figure 5A; Figure S3A). Indeed, the low incidence means some simulations show no infection in a village. The WTP required to carry out active screening is also lower for Mosango, shown by the cost-effectiveness acceptability frontier (Figure S3B), but for $WTP_C > 0.02$, annual active screening is the cost-effective strategy in terms of both the cost-effective acceptability frontier and cost-effective acceptability curves.

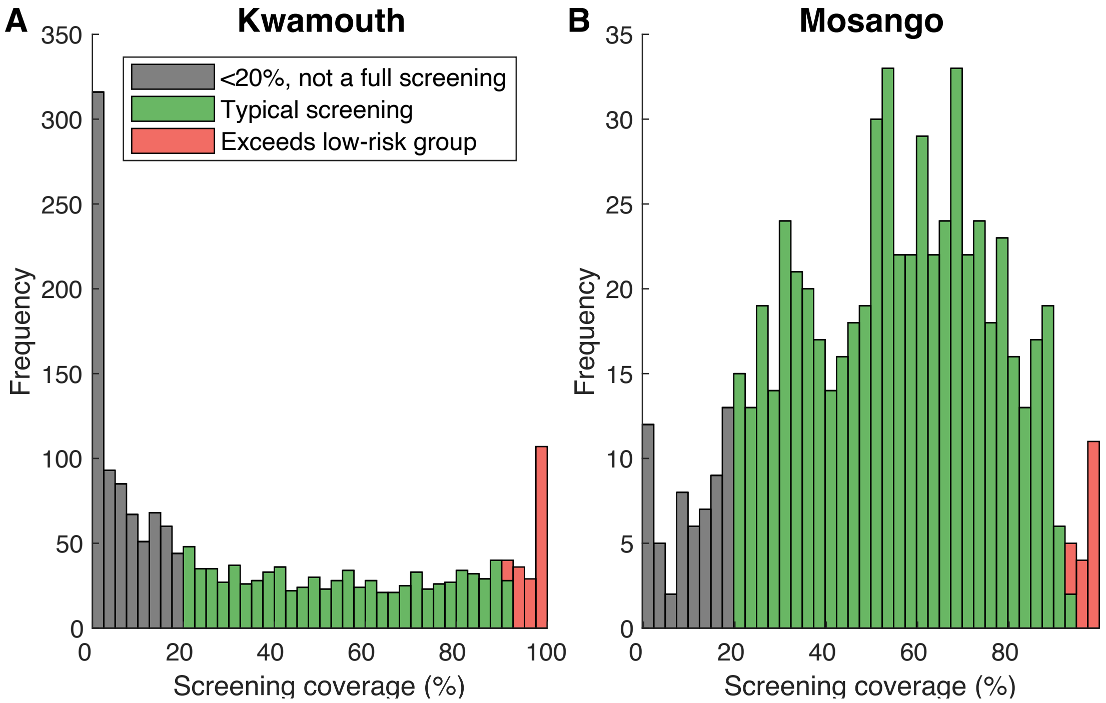

Figure S2: Histograms of screening coverage for (A) Kwamouth and (B) Mosango. We show the coverages below 20% in grey and above the full low-risk group population screened in red, with typical screening coverages shown in green.

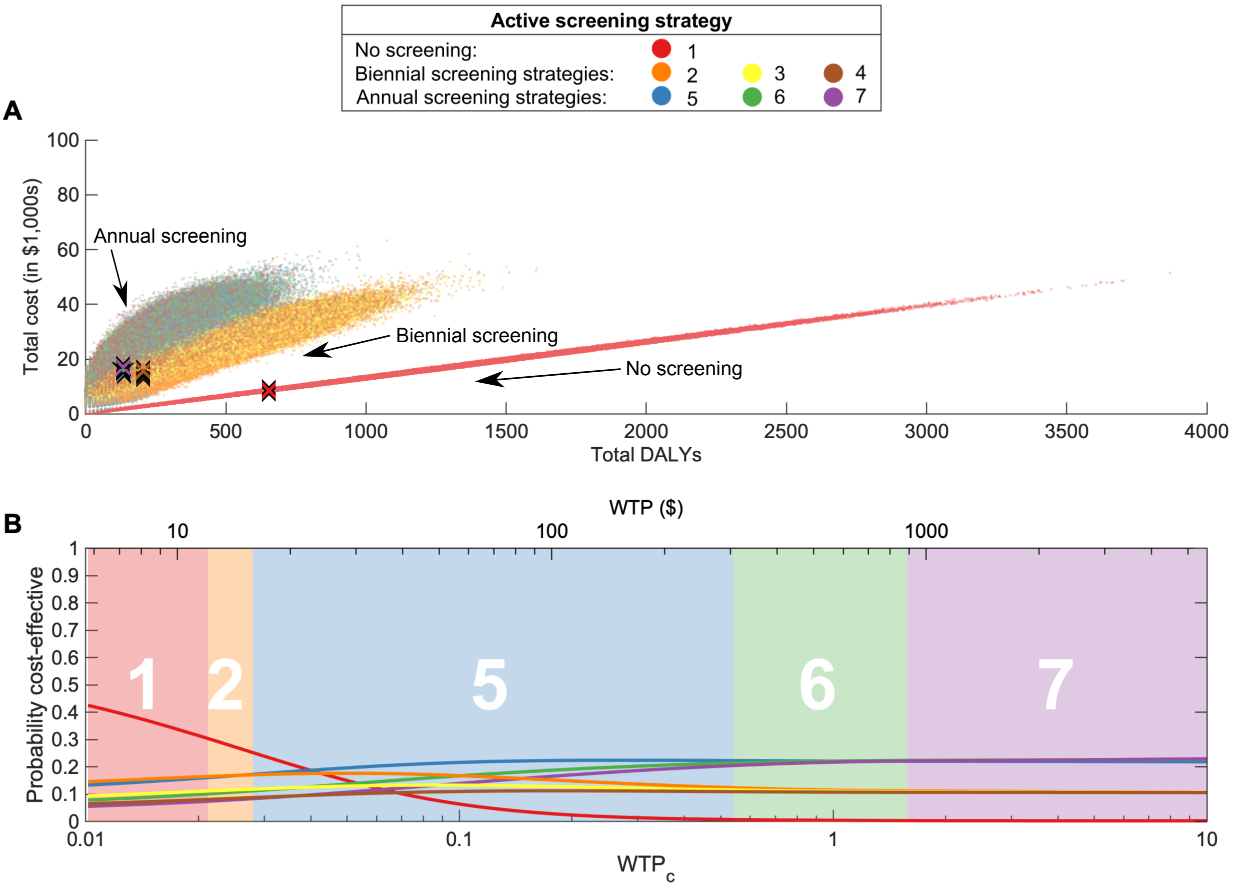

Figure S3: The cost-effectiveness of active screening strategies for the health zone of Mosango. (A) Cost-effectiveness plane showing the total cost of a strategy and the associated total number of DALYs averted from the mean value of the comparator strategy. (B) Cost-effectiveness acceptability curves (CEACs) for each strategy are shown by lines, with the cost-effectiveness acceptability frontier (CEAF) shown by the numbered background colour, which demonstrated the values for the ICER. WTP is shown in 2018 USD on the top and as the WTP_c coefficient on the bottom, where the coefficient is the multiplier of the GDP per capita of the DRC.

Reactive screening

In the main manuscript, we have only considered a scenario where one reactive screening with no cases detected is required to stop screening again $z_r = 1$. However, to be more certain that there is no infection, more reactive screenings may be required. By considering when $c = 90\%$, $t = 1$ year, and $z_a = 3$, we calculate the probability of being cost-effective for the four strategies of no active screening and $z_r = 1, 2, 3$ (Figure S4). Similarly to Figure 5B, a low WTP of less than 3% of the GDP per capita of the DRC means no active screening has the highest probability of being cost-effective, and for higher WTP, active screening is beneficial. The effect of z_r is, however, very minimal, with the chosen three values of z_r giving almost equal probability, although $z_r = 1$ has the highest probability and $z_r = 3$ the lowest.

The limited effect of z_r is explained by the small probability that reactive screening occurs at all (Figure S5A). When $z_a = 3$ and three consecutive zero-detections have occurred, the probability of continuing to find cases to restart screening is small, approximately 5%. Secondly, if reactive screening does occur, it will occur a long time in the future, where discounting has more of an effect and hence the differences in z_r will be negligible.

We observe when $z_a = 1$ the expected number of screenings will vary a little depending on z_r, as reactive screenings are likely to be required, but the variance decreases for larger values of z_a (Figure S5B). Indeed, when looking at the number of screenings that were unnecessary, with full information of the infection (since there was actually no infection), there are more unnecessary screenings when there are more active zero-detections; more reactive zero-detections only have a significant impact when $z_a = 1$ and 2 (Figure S5C).

Hence, we cannot say much about the optimum number of reactive screenings when considering the NMB. The model does not directly consider the benefit of extensively screening to achieve local elimination in a village and there is little differentiation in the values for z_r.

Also, an important factor to determine the reactive screening strategy is the span of time between the detection of a passive case when there is no active screening in the village and the initiation of reactive screening. We have assumed in the main manuscript that reactive screening restarts immediately, although we recognise there would be some delay. However, we show that there is limited effect of this time interval, except for when the number of

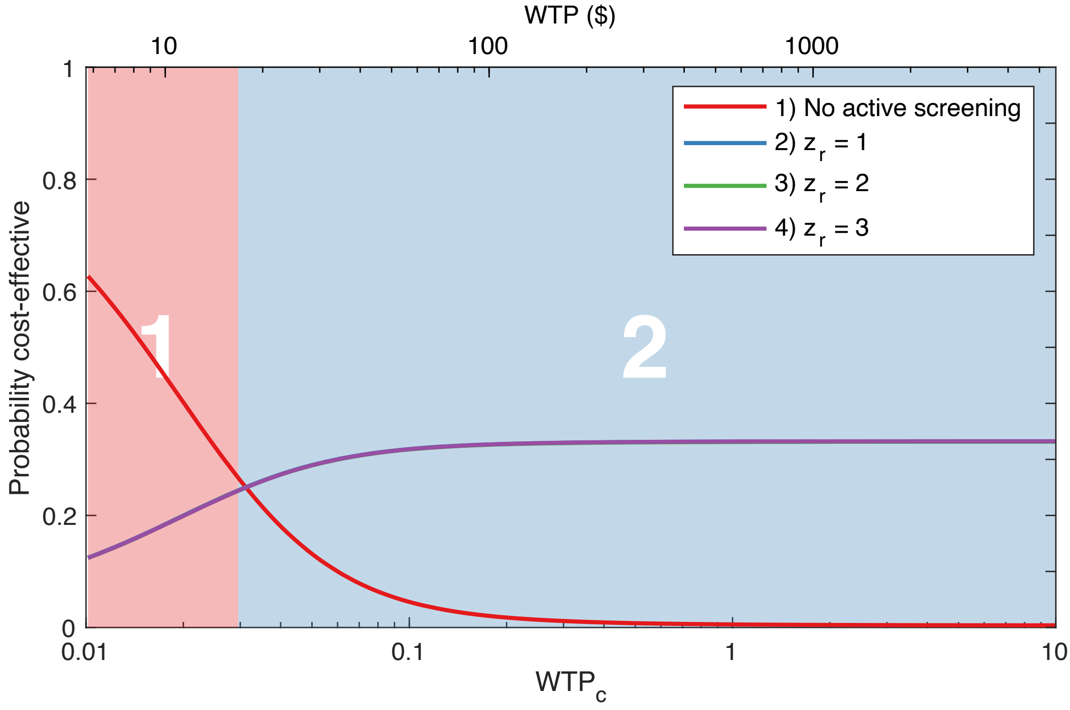

Figure S4: The effect of the number of zero-detections in reactive screening. Probabilities that a reactive screening strategy is the most cost effective (CEACs) are shown by the colored lines (strategies with screening overlap) and the cost-effectiveness acceptability frontier is shown by the coloured background. We assume $c = 90\%$, $t = 1$, and $z_a = 3$ and the population is of size 1,000 starting from endemic equilibrium with $p_t = 27\%$.

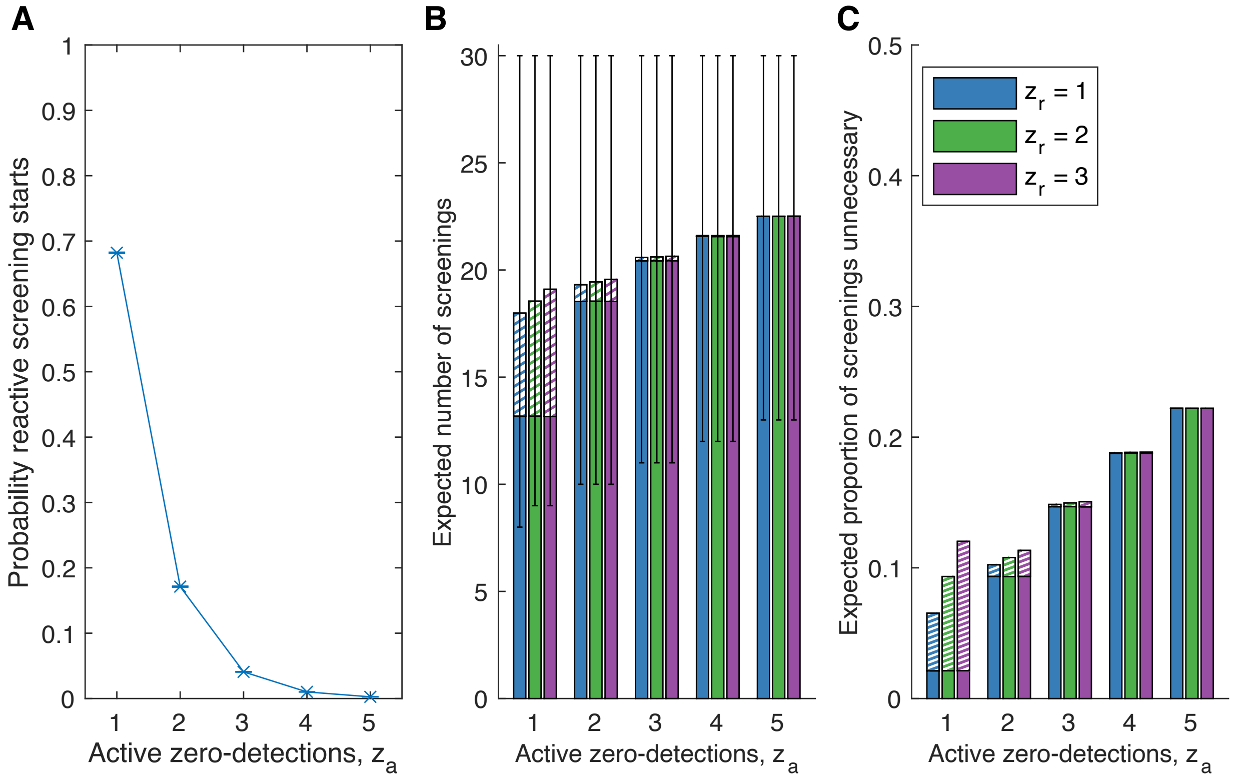

Figure S5: Active and reactive screenings. (A) The probability that reactive screening starts for different z_a values. (B) The expected number of screenings. Active screening are solid bars, while striped bars are reactive screenings. The colour of the bar indicates the value of the reactive zero-detections. 95% prediction intervals are shown. (C) The expected proportion of screenings where there is actually no infection in the village. Sufficient simulations have been run such that confidence intervals are too small to plot.

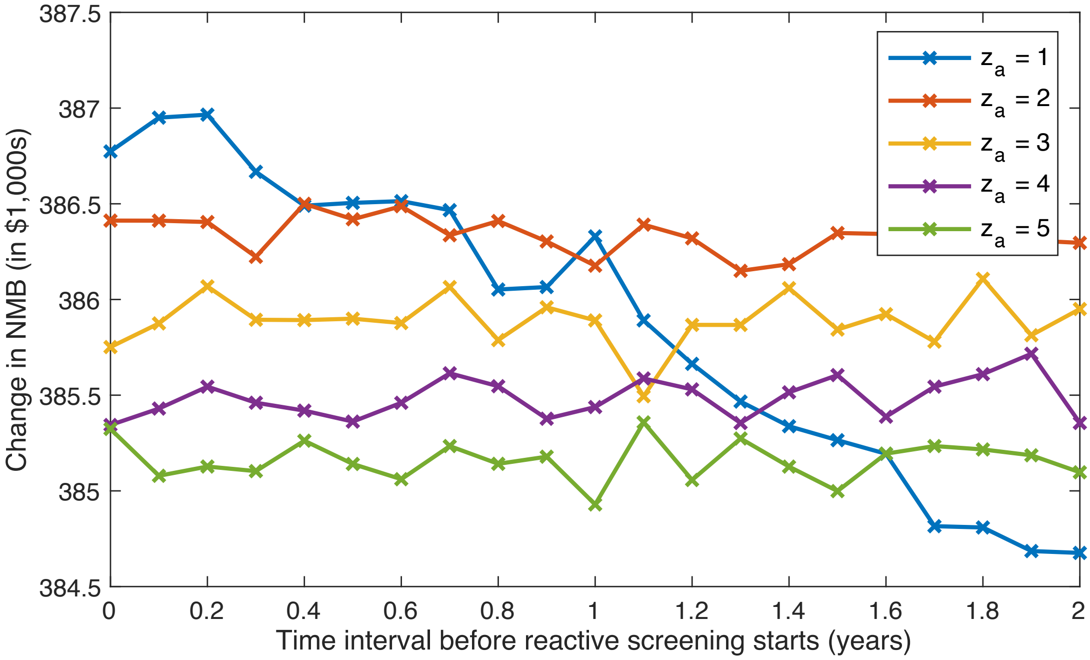

Figure S6: The time interval between observing a passive detection and resuming reactive screening for different values of active zero-detections z_a.

active zero-detections is small (Figure S6). When $z_a = 1$, there is only one active zero-detection required to stop active screening, and so it is very possible there is still infection despite the absence of observed cases on a particular screening. In other words, when the time interval for reactive screening is small, reactive screening can resume very quickly upon a false zero-detection. We see the effect of a delay in reactive screening slightly reduces the total NMB for $z_a = 1$ and for $z_a = 2$, with any effect of higher z_a values completely outweighed by the stochasticity of the epidemic process. We note that in practice, there would be some delay before reactive screening begins, but the difference in NMB is small, less than a $1,000 difference for delays between 0 and 1 years for $z_a = 1$, the value of z_a that is most affected.

Additional results

Time-horizons

In all simulations in the main manuscript we have used a time-horizon of 30 years, since this is sufficient to capture the majority of the costs and DALYs of an active screening strategy on a village until cessation. Figure S7 shows the distribution of the change in costs and the number of DALYs averted when the simulation is run for both 30 and 100 years using a 55% screening coverage annually with three zero-detections in a population of 1,000 starting from endemic equilibrium with 27% of passive infections treated ($c = 55\%$, $t = 1$ year, $z_a = 3$, $N_H = 1,000$, $p_t = 27\%$). The distribution is broadly identical since only a minimal amount of cost incurred after the 30 years. Indeed, we see that in 94.3% of the simulations no additional costs or DALYs are incurred by expanding the time-horizon to 100 years. The mean cost for 30 years is 99.1% of the mean cost for 100 years, and the mean DALYs incurred in 30 years are 99.8% of those incurred over 100 years. Hence, we are satisfied using a time-horizon of 30 years.

Heatmaps of the theoretical optimum

We calculated the optimal strategy using the mean infection dynamics for specific p_t values in Figure 3. Here, we show heatmaps for the optimal strategy across all p_t values, shown on the y axes (Figure S8A–C). We also show the associated NMB of the optimal strategy and breakdown between the cost of the strategy and the number of DALYs averted (Figure S8D–F).

The effect of different treatments on the NMB

In the main manuscript, we used fixed cost and benefit parameters for all simulations. These costs are liable to change over time and have the potential to change dramatically if new practices are implemented, such as a change in treatment. Thus, we have a supplementary R Shiny [58] web app at https://christopherdavis.shinyapps.io/optimising-ghat-active-screening/. A selection of figures can then be generated for any updated costings for the active screening strategies.

In particular, we show how the change in costs would change in four different scenarios: CATT screening tests were replaced by RDT; capillary tube centrifugation (CTC) was additionally used in the microscopy procedure; all treatment was carried out using pentamidine for Stage 1 and NECT for Stage 2; all treatments are replaced by

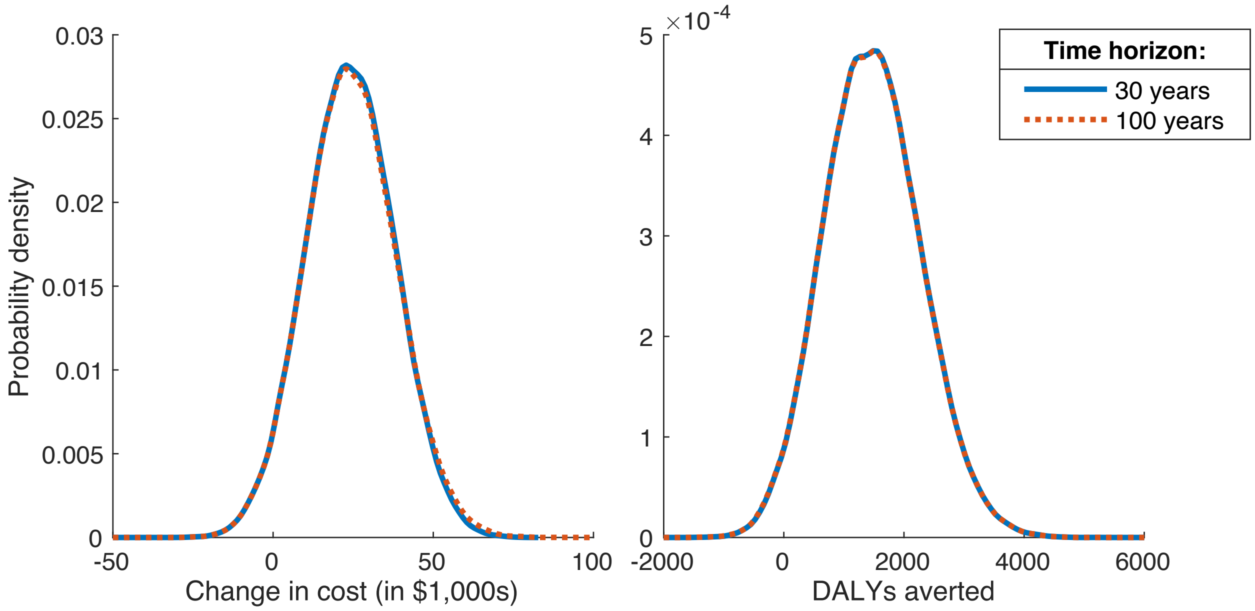

Figure S7: Distributions of (A) the change in cost of an active screening strategy and (B) the number of DALYs averted for 30- and 100-year time-horizons. The simulation is for 55% screening coverage annually with 3 zero-detections in a population of 1,000 starting from endemic equilibrium with 27% of passive infections treated.

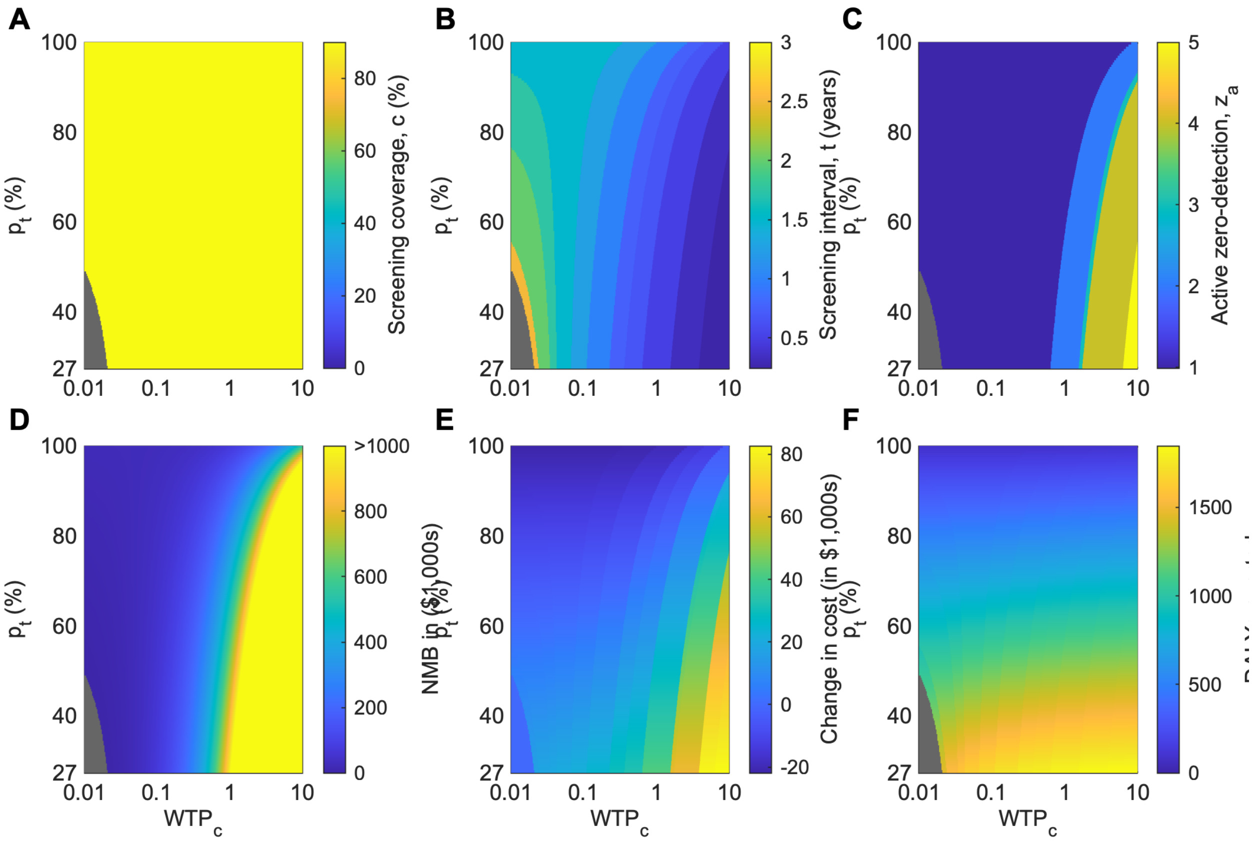

Figure S8: Theoretical optimum strategy for mean simulation of infection dynamics given WTP and p_t. (A-C) The screening coverage, screening interval and number of zero-detections required to stop screening (respectively) that achieves the highest NMB. (D) The NMB of that strategy. (E) The change in cost. (F) The number of DALYs averted. We use a village population of 1,000, where the disease in endemic. The plots are grey where 'no active screening' is the optimal strategy.

fexinidazole (Figure S9). The assumed increased cost of RDT compared to CATT impacts the total change in cost most significantly as it would be applied to all those undergoing active screening. The effect of CTC in microscopy is negligible when considering a whole village for 30 years.

Changing the treatments will also not greatly change the costs since the total treamtent cost is not the largest component cost. However, replacing the other treatments with only fexinidazole, and therefore reducing the cost of treatment, will counter-intuitively increase the difference in the total cost between the active screening programme and the comparator strategy (no active screening). The active screening strategy means that there will be fewer total infections treated, thus a reduced treatment cost will mean the total treatment cost will be less cost-saving overall.

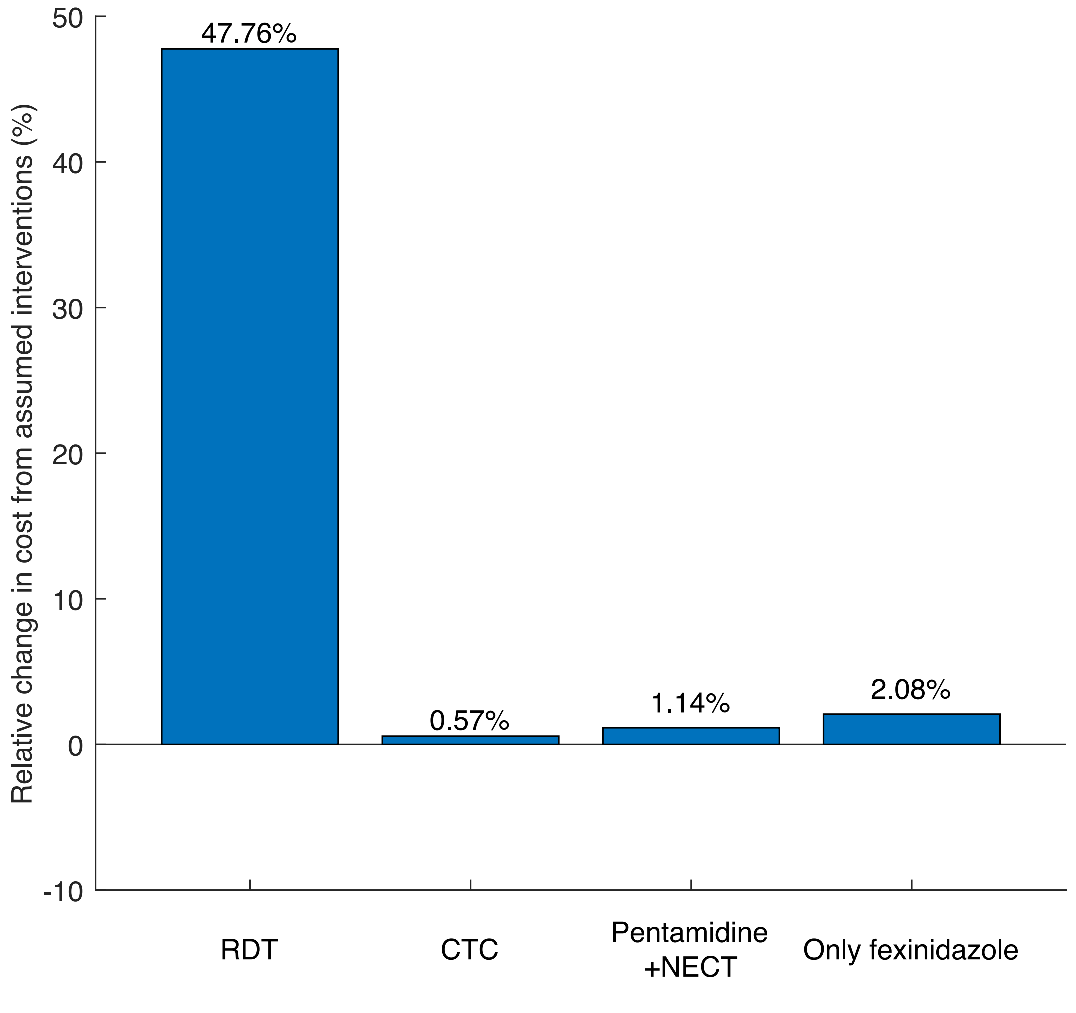

Figure S9: Mean change in costs relative to the assumed interventions for a village with 55% screening coverage annually with 3 zero-detections. We consider the replacement of CATT with RDT, the addition of CTC in microscopy and the use of fexinidazole in Stage 2 patients and all patients. We use a population of 1,000 starting from endemic equilibrium with 27% of passive infections treated.

Differences in active screening strategies

We fit a linear regression through the strategies from Figure 5. The gradient and slope is very similar for strategies with the same screening interval and very different between them. This indicates that the costs and DALYs averted are very different for the different screening intervals but no large differences exist for alternative values of active zero-detections. The values of the linear fits are shown in Figure S10 and Table S6.

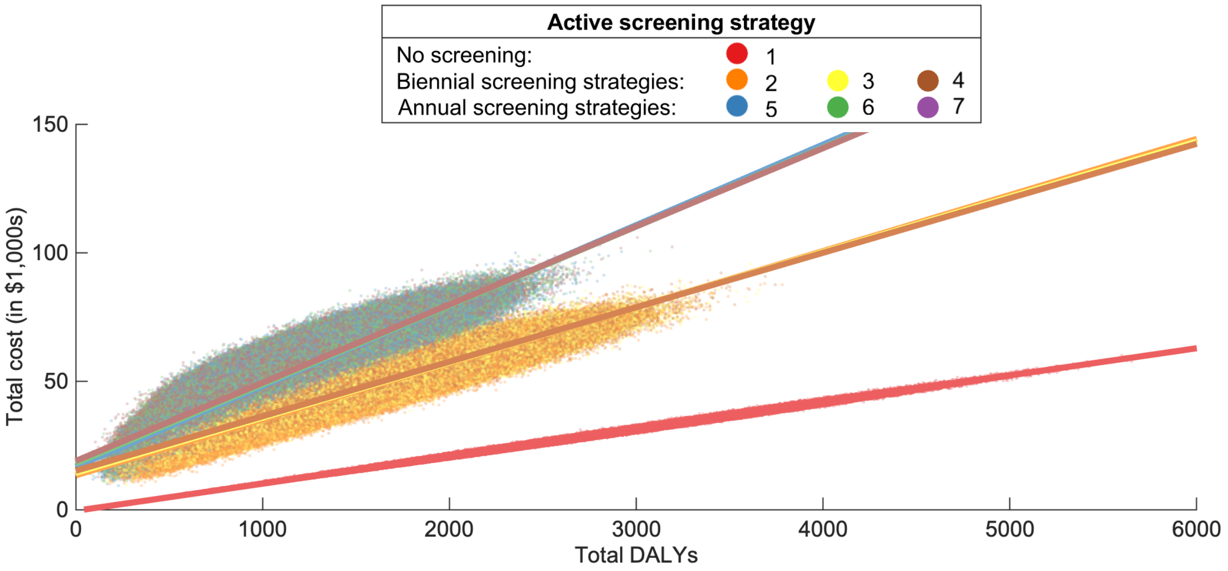

Figure S10: Linear fits through the results of different active screening strategies from Figure 5. Full detail in Table S6.

Table S6: The values that determine the linear fits for each strategy in Figure S10.

Strategy	Gradient	Intercept
1	0.0105	-0.5
2	0.0218	13.3
3	0.0215	14.3
4	0.0212	15.1
5	0.0313	16.7
6	0.0307	18.0
7	0.0304	18.9

The five principles of the Neglected Tropical Diseases Modelling Consortium

We have detailed how we communicated our model to stakeholders in line with the framework set out by the NTD Modelling Consortium (Table S7) [59].

Table S7: PRIME-NTD criteria fulfillment. How this study has satisfied the five principles of the Neglected Tropical Diseases Modelling Consortium.

The principle and what has been done to satisfy this principle?	Where in the manuscript is this described?
**1. Stakeholder engagement**	
The study was led by modellers with guidance from E Mwamba Miaka of the national sleeping sickness control programme in DRC (PNLTHA-DRC). PNLTHA-DRC have provided detailed knowledge of the data and information as to how the programme has changed over time.	Authorship list
**2. Complete model documentation**	
Original deterministic model code used for fitting, and the stochastic model code described here is available on OpenScienceFramework (OSF) at https://osf.io/msbnq/. The model is fully described in the manuscript and the appendix.	OSF, Materials and Methods, Appendix
**3. Complete description of data used**	
To parameterise our model we used the median of the distributions inferred using MCMC methodology with aggregate annual data from the WHO HAT Atlas in Kwamouth and Mosango [60]. This data used to fit the models and the fitting process is described in detail in Crump *et al.* [30].	Materials and Methods, Franco *et al.* [60], Crump *et al.* [30]
**4. Communicating uncertainty**	
Structural uncertainty: The structure of the model was determined in a model fitting exercise with other plausible structures compared [22]	Materials and methods, Rock *et al.* [22]
Model parameter uncertainty: The model was fitted using MCMC methodology with data specific to the health zones considered. Model simulations were done using the median value of these fitted posterior distributions.	Material and methods, Crump *et al.* [30]
Cost/benefit parameter uncertainty: Our best estimate for each cost/benefit parameter is given with details of how we calculated this value. The accompanying webapp also allows cost parameter values to be modified by the user, with the resulting outputs displayed (https://christopherdavis.shinyapps.io/optimising-ghat-active-screening/).	Appendix, webapp
Stochastic uncertainty: All stochastic model realisations were repeated one million times with the mean value computed (where appropriate), such that the confidence interval of the mean value was negligible and not visible when plotted. The range of cost and DALY outcomes is also displayed in a cost-effectiveness plane.	Figures
**5. Testable model outcomes**	
Linear fits are performed for the model results for the different strategies to show that the screening interval has a much larger impact on the outcomes than the active zero-detections, in agreement with the cost-effectiveness analysis.	Figure S10 and Table S6
